# Annotations

**Published:** 1897-01-02

**Authors:** 


### Annotations.

### House Warranty.

Since the particular "environment" which influences our health almost more than any other is the house we live in, every legal case which tends either to clear up the present state of the law with regard to the "warranting" of the sanitation of houses, or to show the necessity for some improvement in the law, is of great importance. The case of Green v. Symons, tried last week before Mr. Justice Lawrance, is very simple, but as instructive as it is simple. The statement of plaintiff's claim shows that Miss M. P. Symons verbally "warranted" a house, known as Earlswood House, at Earlswood, in 1894, as being "perfectly dry, and the drains in perfect order." On this warranty Mr. W. H. Green became tenant of the house on a three years' agreement. Mr. Green found, on residence, that the house was not dry, and that the drains were not good. Indeed, so bad was the condition of things, that he left the house, and sued Miss Symons for damages. In the result, a verdict for £50 damages was given in his favour. But the opposing counsel objected to the verdict on an interesting point. He did not dispute the fact of the "verbal warranty," but he contended, that as there was a written agreement between the parties, and that as the warranty was not included in the written agreement, it was of no binding validity. The judge did not allow this contention to stand; but he gave leave to appeal, and there the question is for the time suspended. But there is no need to wait for the decision of the Court of Appeal in order to deduce the moral of the case. The moral is this: that a tenant should on no account take a house without having a "warranty" of perfect sanitation inserted in his written agreement; and if there be no written agrement, then a written warranty should be asked for. One cannot help wondering at the carelessness—or is it stupidity?—of the British householder in his character of a tenant. The stupidest lout in a country fair will ask for a written warranty if he buys a horse. How much more important to the health and happiness of the family is a house than a horse? The only change, apparently, that is required in the law is to make written warranties compulsory on the part of landlords.

### Richard III.—A Medico-Psychological Study.

In studying psychology, medicine goes to the living human subject, to the lunatic asylum, to the bed-side of the neurotic, to the daily life of the developing youth and maiden, and of the fully developed and healthy man and woman rather than to books. As a study of intense psychological interest from the medical standpoint, there are few historical personages who can be compared with Richard III., that "dreadful portent of the House of York." Richard, though undoubtedly deformed, must have been a man of tremendous physical energy, as deformed people of the criminal type not seldom are. His mind was of the order that sees beforehand, and "wills the end." "He who wills the end wills the means," it is said. Richard, as Sir Henry Irving shows, made no hesitation about the means. His murders of the little princes, his love passages, first with Anne and then with Elizabeth, his behaviour towards his associate, Buckingham, his determination to sweep away, as if without a moment's hesitation, or even thought, friends, enemies, relations, and strangers, whoever stood between him and the seizing and holding of the crown—these show evidences, as we should now think, of a monster rather than a man. The average man thinks all such things impossible in modern times; thinks types like Richard are totally extinct. The medical psychologist knows better. He can put his finger upon many a potential murderer who has never murdered; upon men and women who would wade through seas of blood to gain their ends, if by so doing the ends could with certainty be gained and a just retribution avoided, even for a time. The medical psychologist, in a word, knows well that human nature has undergone no essential change since the days of Richard, or even since the days of Pharaoh. Education has modified manners—has made men more apprehensive of consequences, has improved the character of the majority, and so has placed restraint upon the criminal types, and made crime both more difficult and less profitable. Law has stepped in and strengthened the hands of education. But human nature remains the same; and those who wish to see the dreadful possibilities which lie open to the human kind, some of which possibilities may even lie open to the very best, should not fail to visit the Lyceum whilst Richard III. is on the boards.

### Female Mortality in the Liquor Trades.

Some four or five years ago a committee was appointed, of representatives of some of the principal life assurance offices, to investigate the question of the relative mortality of persons engaged in the sale of intoxicating drinks as compared with the mortality of similar persons not so engaged. Among some of the "unexpected facts" brought to light by the investigation was this—that "female publicans and female grocers show a very favourable rate of mortality." As compared with male innkeepers and male grocers the difference is striking; so much so, that whilst a very considerably larger premium than ordinary is demanded by all life offices from all male assurers belonging to the publican class, and the class of wine and spirit selling grocers, a premium almost identical with that of ordinary assurers is found to cover the risk in the case of females engaged in the same occupations. Curiously enough, the wives of male publicans and of male grocers who sell wines and spirits are found to be more liable to disease and death from alcoholism than the females who follow the liquor business independently. It is not easy to explain these circumstances, except on the assumption that, in the first place, women are naturally more disposed to sobriety and good conduct than men; and that, secondly, such women as are willing to play an independent part in the world possess a strength of character which is not shown so decisively by those who depend upon husbands or other male protectors. It might have been supposed that women, in consequence of their greater sensitiveness, would, when not under male control, be much disposed to give way to the temptations of alcoholism. It is not so, however, by any means among women who are really capable; and the facts, so far as they go, offer distinct encouragement to such to adventure boldly upon the sea of open competition, and to trust to their superiority in certain branches of morals and conduct to bring them success.

